# Effect of Health Literacy on Quality of Life amongst Patients with Ischaemic Heart Disease in Australian General Practice

**DOI:** 10.1371/journal.pone.0151079

**Published:** 2016-03-04

**Authors:** David Alejandro González-Chica, Zandile Mnisi, Jodie Avery, Katherine Duszynski, Jenny Doust, Philip Tideman, Andrew Murphy, Jacquii Burgess, Justin Beilby, Nigel Stocks

**Affiliations:** 1 Discipline of General Practice, School of Medicine, NHMRC Centre of Research Excellence to Reduce Inequality in Heart Disease, The University of Adelaide, Adelaide, SA, Australia; 2 Population Research and Outcome Studies, Discipline of Medicine, School of Medicine, The University of Adelaide, Adelaide, SA, Australia; 3 Discipline of Paediatrics, School of Medicine, The University of Adelaide, Adelaide, SA, Australia; 4 Faculty of Health Sciences and Medicine, Bond University, Robina, QLD, Australia; 5 Department of Cardiovascular Medicine, Flinders University, Adelaide, SA, Australia; 6 Department of General Practice, National University of Ireland, Galway, Ireland; 7 Centre for Children's Burns & Trauma Research, Child Health Research Centre, University of Queensland, Brisbane, QLD, Australia; 8 Vice-Chancellor, Torrens University Australia, Adelaide, SA, Australia; National Institute of Genomic Medicine, MEXICO

## Abstract

**Background:**

Appropriate understanding of health information by patients with cardiovascular disease (CVD) is fundamental for better management of risk factors and improved morbidity, which can also benefit their quality of life.

**Objectives:**

To assess the relationship between health literacy and health-related quality of life (HRQoL) in patients with ischaemic heart disease (IHD), and to investigate the role of sociodemographic and clinical variables as possible confounders.

**Methods:**

Cross-sectional study of patients with IHD recruited from a stratified sample of general practices in two Australian states (Queensland and South Australia) between 2007 and 2009. Health literacy was measured using a validated questionnaire and classified as inadequate, marginal, or adequate. Physical and mental components of HRQoL were assessed using the Medical Outcomes Study Short Form (SF12) questionnaire. Analyses were adjusted for confounders (sociodemographic variables, clinical history of IHD, number of CVD comorbidities, and CVD risk factors) using multiple linear regression.

**Results:**

A total sample of 587 patients with IHD (mean age 72.0±8.4 years) was evaluated: 76.8% males, 84.2% retired or pensioner, and 51.4% with up to secondary educational level. Health literacy showed a mean of 39.6±6.7 points, with 14.3% (95%CI 11.8–17.3) classified as inadequate. Scores of the physical component of HRQoL were 39.6 (95%CI 37.1–42.1), 42.1 (95%CI 40.8–43.3) and 44.8 (95%CI 43.3–46.2) for inadequate, marginal, and adequate health literacy, respectively (p-value for trend = 0.001). This association persisted after adjustment for confounders. Health literacy was not associated with the mental component of HRQoL (p-value = 0.482). Advanced age, lower educational level, disadvantaged socioeconomic position, and a larger number of CVD comorbidities adversely affected both, health literacy and HRQoL.

**Conclusion:**

Inadequate health literacy is a contributing factor to poor physical functioning in patients with IHD. Increasing health literacy may improve HRQoL and reduce the impact of IHD among patients with this chronic CVD.

## Introduction

Ischaemic heart disease (IHD) is a common cardiovascular disease (CVD), and a leading cause of global morbidity and mortality [1]. In Australia, more than a half million adults live with IHD, and as the principal cause of mortality was responsible for more than 20,000 deaths in 2011 [2]. Treatment improvements of acute cases were responsible for a 4% per year mortality rate decline in the last three decades. Nevertheless, frequent complications and hospitalisations related to this condition are expected to increase total health expenditures from 9.4% in 2002–03 to 10.8% in the next 30 years [3]. Given this resource burden, the contemporary management of IHD and other CVD should focus not only on decreasing morbidity and mortality, but also on improving quality of life (QoL) [4–6]. QoL is directly affected by CVD and other chronic conditions, and represents a good indicator of patients’ functional status, as physiological measures provide limited information on individual’s capacity to engage in daily activities [4, 7].

The chronic nature of IHD requires commitment to prolonged therapy [8] and sufficient understanding of health information by patients to enable them to participate actively in the management of their health condition [9]. Health literacy is defined as the “cognitive and social skills possessed by the individual that enables them to appropriately access, understand and use health information to maintain good health”[10]. Therefore, it is a critical component of comprehensive patient care [11]. Low health literacy has been observed to be a problem not only in low-and-middle income countries but also in affluent societies. In Australia, it is estimated that only 40% of adults have a basic level of functional health literacy, although approximately 70% of all adults have an educational attainment equivalent to a high school or better educational level [12, 13].

Scientific literature suggests that inadequate health literacy adversely affects health outcomes [9, 11, 14], medical costs [15] and health-related QoL (HRQoL) [9, 16, 17]. Disease knowledge, behavioural changes, and a more active involvement in the management of their health condition are factors that can influence HRQoL in patients living with chronic conditions [9, 17]. Therefore, optimal health literacy seems critical to improving QoL and health status, especially when targeted primary and secondary preventive strategies struggle to achieve expected levels [18, 19].

Few studies have examined the effects of health literacy on HRQoL among patients with CVD [17, 20] and these have been limited to a focus on heart failure. Although these studies provide insights into this topic, it is important to document health literacy levels among individuals living with other CVDs to evaluate possible differences. Additionally, these studies used disease-specific tools to measure HRQoL [17, 20], which have been criticised for missing the overall impact of the disease on general functioning as a result of their tendency to focus on clinical correlates of the disease [21]. These methodological characteristics compromise their comparability, as well as the external validity of the results. Finally, the influence of educational level, socioeconomic conditions, and other demographic variables into this association has not been sufficiently evaluated. Thus, the purpose of this paper is to explore the effect of health literacy on two different domains of HRQoL (physical and mental components) in adult patients living with IHD, and to investigate the role of sociodemographic and clinical variables as possible confounders in this association.

## Methods

This study obtained ethical approvals from the Human Research Ethics Committees associated with the University of Adelaide and the University of Queensland. All the participants signed the respective consent term.

To investigate the association between health literacy and HRQoL this cross-sectional study analysed data on patients with IHD recruited from general practices in two Australian states (Queensland and South Australia) between 2008 and 2009.

### Participants

Lists of general practices were obtained from both states in 2007. Practices were stratified according to the number of general practitioners (GPs) (1–2, 3–5, or 6+ GPs) and location (urban or rural). Using a systematic sampling process in each strata, a total of 24 practices were sequentially recruited between 2007 and 2008 (33% with 1–2 GPs, 46% with 3–5 GPs, and 21% with 6+ GPs; 71% in urban areas). Patients with IHD were then identified from the general practice clinical databases using a validated overseas protocol [22], modified to include other diagnoses (“myocardial infarction” and angina), procedures (“coronary angioplasty” and “coronary artery bypass grafting”), and their synonyms. These terms were combined with prescribed medications (nitrates, aspirin, atenolol, digoxin, and statins) to improve search sensitivity. Given the estimated prevalence of IHD in general practice (2.5–5.0%), it was expected there would be a target population of 1,600–3,000 patients in the recruited general practices [23]. Lists were reviewed by their GPs, with patients excluded if terminally ill, having dementia (or other significant cognitive impairment), unable to speak and/or write in English, no longer associated with the practice, or for any other reason at the discretion of the GPs. Thus, after this process the final target population was 2,132 patients with IHD.

Each practice/GPs sent the first of two questionnaires to this list of potentially eligible patients, together with a letter from the practice endorsing the study and seeking approval to audit the patient’s clinical notes. A total of 812 individuals returned the first questionnaire. A second questionnaire was sent to these responders one month after receipt of the first to limit questionnaire fatigue (including questions on HRQoL). A total of 587 patients with IHD returned the second survey (72.3% of respondents to the first questionnaire), which is the final sample size of this study. Sociodemographic variables collected in these questionnaires included: age, gender, educational level, perceived socioeconomic status, and postcode area of residence in which the individual lived most of the time in the past 12 months. Based on the last information, the Australian Socio-Economic Indexes for Areas (SEIFA) Index of Relative Socio-economic Advantage and Disadvantage (IRSAD) was used to provide a measure of socioeconomic status divided in quintiles (highest, high, middle, low, and lowest). This index summarises information about the economic and social conditions of people and households within an area, including both relative advantage and disadvantage measures [24]. Problems recruiting GPs in lower socioeconomic areas resulted in a lack of patients with IHD belonging to the lowest SEIFA-IRSAD quintile.

The questionnaires also evaluated participants’ general health characteristics, collecting data on risk factors for cardiovascular diseases (self-reported weight and height, alcohol consumption, smoking, nutritional habits, physical activity, depression, anxiety, and stress), health literacy, and HRQoL. Biomedical information was collected from medical records, including type of ischaemic condition (angina or myocardial infarction), history of revascularisation procedures (coronary angioplasty or coronary artery bypass graft), time since first ischaemic episode (IHD diagnosis or revascularization procedure), diagnosis of other CVD, presence of clinical CVD risk factors (hypertension, dyslipidaemia, diabetes mellitus, and chronic kidney disease), use of pharmacological agents, blood pressure, glycaemia, and lipid levels,

### Independent variable

Health literacy was measured using a validated 16 item questionnaire developed by Chew et al [25], which evaluates patients´ ability to read and write health-related material. Each question includes a Likert scale, ranging from 0 (always) to 4 (never), some of them requiring reverse code translation to allow higher scores representing higher health literacy levels. Scores were combined into a total score ranking from 0 to 64 points, and subsequently transformed into categories of health literacy (0–34 = inadequate, 35–42 = marginal, 43–64 = adequate) using equivalent cut-off points to the gold standard instrument used for validation (Short Test of Functional Health Literacy in Adults–STOFHLA) [25, 26].

### Outcome

HRQoL was measured using the Medical Outcomes Study Short Form 12 (SF-12) [27], a 12 item questionnaire that summarizes QoL into two measures: a physical component and mental component [28]. The time reference for all the questions in the SF-12 was the past four weeks. Scores for each of the 12 questions were transformed and combined into 0–100 scales for the physical and mental components, with 100 representing the best HRQoL [27, 29].

### Covariates

Sociodemographic and clinical variables were included as possible confounders of the association between health literacy and HRQoL, and selected based on the literature review [4–6, 17, 20]. Sociodemographic variables included gender (male or female), age (continuous variable and a quadratic term due to nonlinear relationship with HRQoL), marital status (married or unmarried), area of residence (rural or urban, based on postcodes), state of general practice (South Australia or Queensland), and attained educational level (degree or higher; certificate to advanced diploma; secondary; no schooling to primary). Due to the reduced number of individuals who reported no formal education (n = 8), they were included in the same category of primary eduaction to avoid sample size reduction. Working status (employed full or part time; retired/pensioner /receiving benefit; unemployed), perceived economic situation compared to people of the same age (very high/higher; similar to other people; lower/very low), and economic position (quintiles of SEIFA-IRSAD) were also evaluated as sociodemographic variables. Clinical variables included history of revascularisation procedures (yes/no), years since first IHD episode (up to 5, 6–10 and 10+ years), and number of other CVD. The total number of other CVD conditions (atrial fibrillation/another arrhythmia, heart failure, stroke, heart valve disease, heart-block, cardiomyopathy, and left-ventricular dysfunction) was computed and then transformed into an ordinal variable (0, 1, and 2+ conditions). Diagnosis of hypertension, dyslipidaemia, diabetes mellitus, chronic kidney disease, and overweight (BMI ≥25 kg/m², using self-reported data on weight and height) was used to estimate the number of CVD risk factors and then treated as an ordinary variable (0–1, 2, and 3+ risk factors).

### Power of the study

The power of the study was estimated *a posteriori* considering the final sample size of 587 individuals with IHD. For a prevalence of inadequate health literacy of 14.3%, and fixing the alpha in 5% and the power in 80%, it would be possible to detect a mean difference of at least 3.6 points for the physical component of HRQoL (for a standard deviation = 10.9) and 2.7 points for the mental component (for a standard deviation = 7.9).

### Data analysis

Descriptive analyses were performed considering absolute and relative frequencies (%) for categorical variables, and mean with their standard deviation or median with interquartile range (p25-p75) for numerical variables. Confidence intervals of 95% (95%CI) were also estimated. Health literacy was treated as a dichotomous variable (inadequate or marginal/adequate health literacy) to evaluate its association with sociodemographic and clinic variables. Chi-square tests for heterogeneity or trend were used. When the HRQoL scores were evaluated, bivariate analyses were performed with t-test or ANOVA (heterogeneity or trend test) depending on the nature of the independent variables.

Multivariate linear regression was used to evaluate the association between health literacy (as a polytomous variable with three categories) and HRQoL. Regression coefficients (β) with their respective 95%CI were estimated in crude and adjusted analysis, using adequate health literacy as the non-exposed group. A p-value <0.05 was considered as indicative of statistical significance. Sociodemographic and clinical variables were treated as possible confounders, and manually included in the models using a forward selection process when the β on the outcome showed a level of significance <0.10. Additionally, the determination coefficient (r^2^) was used to evaluate the overall model fit. Finally, the variance inflation factor (VIF) was investigated as an indicator of multicollinearity between the explanatory variables. All the analyses were performed in the STATA software, version 13.0 (StataCorp, Texas, USA).

## Results

A total of 587 patients with IHD and complete information on HRQoL were recruited from the 24 general practices. Table 1 summarizes some clinical characteristics of the participants. Angina was more common than myocardial infarction, and 71.9% (95%CI 68.1–75.4) of the patients underwent some revascularization procedure. The median time since the first IHD episode was 8.1 years (p25-p75 4.4–13.7). Atrial fibrillation/arrhythmia was the most common CVD comorbidity (55.4%). The median number of clinical risk factors for CVD was 2 (p25-p75 2–3), with hypertension as the most frequent factor.

**Table 1:** Prevalence of cardiovascular conditions, revascularization procedures, and clinical risk factors for cardiovascular disease (CVD) among patients with ischaemic heart disease (N = 587)^a^.

	Prevalence (%)	95%CI
**Ischaemic heart disease**		
Angina	60.1	56.1–64.0
Myocardial infarction	46.7	42.7–50.8
**Revascularization procedure**		
Coronary angioplasty (with or without stent)	41.3	37.4–45.4
Coronary artery bypass graft	39.9	36.0–44.0
**Other CVD conditions**		
Atrial fibrillation or other arrhythmia	16.9	14.0–20.1
Heart valve disease	8.9	6.8–11.5
Heart-block	8.7	6.7–11.3
Stroke	8.6	6.6–11.2
Cardiomyopathy	8.5	6.5–11.1
Heart failure/Congestive cardiac failure	7.9	6.0–10.4
Left-ventricular dysfunction	2.7	1.7–4.4
**Clinical CVD risk factors**		
Hypertension	79.3	75.8–82.4
Overweight (including obesity)*	70.6	66.7–74.3
Dyslipidaemia**	53.3	49.3–57.4
Diabetes mellitus	20.9	17.7–24.3
Chronic kidney disease	3.6	2.3–5.4

a–Percentages does not sum 100%, as some patients had more than one condition

* Body mass index ≥25.0 kg/m², based on self-reported information on weight and height

** Including hypercholesterolaemia, hypertriglyceridaemia, and familial hyperlipidaemia

The mean age of the participants was 72.0±8.4 years, mostly male (76.8%), married (75.1%), living in urban areas (72.9%), with secondary educational level (40.7%), retired or pensioner (84.2%), and with a similar economic situation compared to other people of the same age (62.5%) (Table 2). The proportion of participants in each state and economic position category were relatively balanced. Health literacy showed a mean of 39.6±6.7 points, with 35.2% classified as adequate (95%CI 31.5–39.1), and 14.3% (95%CI 11.8–17.3) as inadequate. Table 2 also shows the distribution of inadequate health literacy according to sociodemographic and clinical variables. No difference was observed according to gender, but the prevalence of inadequate health literacy was three times higher in the oldest (23.1%) when compared to those with <70 years (8.2%). Lower educational level was also associated with a higher prevalence of inadequate health literacy, and although a significant association was also observed with the economic situation, the relationship was non-linear, and again the poorest showed the highest prevalence. The number of CVD comorbidities was the only clinical variable associated with the prevalence of inadequate health literacy (positive trend).

**Table 2:** Bivariate association between health literacy and sociodemographic and clinical variables among patients with ischaemic heart disease.

			Inadequate health literacy^a^		
Variables	N	%	Prevalence %	95%CI	p-value*
**Gender**					
Male	451	76.8	13.6	10.8–17.0	0.361
Female	136	23.2	16.7	11.4–23.8	
**Age group**					
<70 years	199	33.9	8.2	5.1–12.9	<0.001*
70 to 79 years	267	45.5	14.7	10.9–19.4	
≥ 80 years	121	20.6	23.1	16.7–31.1	
**Marital status**^b^					
Married	441	75.1	13.6	10.7–17.3	0.478
Unmarried	146	24.9	16.1	10.9–23.1	
**Residence area**					
Urban	428	72.9	13.1	10.3–16.6	0.153
Rural	159	27.1	17.7	12.5–24.4	
**State of general practice**					
South Australia	329	56.1	15.1	11.7–19.3	0.531
Queensland	258	44.0	13.3	9.7–18.0	
**Attained educational level**					
Degree or higher	64	10.9	9.5	4.3–19.8	0.011**
Certificate to advanced diploma	221	37.7	11.9	8.2–17.0	
Secondary	239	40.7	15.0	10.9–20.2	
No schooling to primary	63	10.7	25.0	15.6–37.6	
**Working status**^b^					
Employed full or part time	93	15.8	11.2	6.3–19.3	0.657
Retired	126	21.5	13.1	8.2–20.1	
Pensioner	368	62.7	14.7	11.4–18.7	
**Perceived economic situation**					
Very high to higher	100	17.0	11.4	6.5–19.2	0.549**
Similar to other people	367	62.5	14.9	11.6–18.8	
Lower to very low	120	20.4	14.4	9.2–21.8	
**Economic position (quintiles)**^c^					
Highest	181	23.3	11.5	7.2–17.8	<0.001**
High	166	31.0	8.7	5.4–13.9	
Middle	119	15.3	13.5	7.9–22.1	
Low	121	30.3	22.5	17.0–29.1	
**History of revascularization procedure**^d^					
No	163	27.8	15.1	10.3–21.4	0.770
Yes	424	72.2	14.1	11.2–17.7	
**Time since first ischaemic episode**^e^					
Up to 5 years	216	36.8	16.2	11.9–21.6	0.933**
6–10 years	179	30.5	9.1	5.6–14.5	
10+ years	192	32.7	16.1	11.6–21.8	
**Number of other CVD conditions**^f^					
0	357	60.8	11.9	9.0–15.6	0.032**
1	147	25.0	17.2	12.0–24.0	
2+	83	14.1	19.5	12.4–29.4	
**Number of clinical CVD risk factors**^g^					
0–1	133	22.7	14.4	9.4–21.4	0.651**
2	215	36.6	15.6	11.4–21.0	
3+	239	40.7	13.1	9.4–18.0	

* Chi-square for heterogeneity

** Chi-square for trend

CVD = cardiovascular disease

a–Score ≤ 34 on Short Test of Functional Health Literacy in Adults

b–Variable with missing data

c–Based on the Socio-Economic Indexes for Areas Index of Relative Socio-economic Advantage and Disadvantaged (SEIFA-IRSAD). Individuals in the lowest quintile were not included due to logistic problems recruiting general practices in poorest areas.

d–Coronary angioplasty or coronary artery bypass graft

e–Angina, myocardial infarction, or revascularization procedure

f—Atrial fibrillation/other arrhythmia, heart failure, stroke, heart valve disease, heart-block, cardiomyopathy, and/or left-ventricular dysfunction

g—Hypertension, overweight, dyslipidaemia, diabetes mellitus, and/or chronic kidney disease

The mean score of the physical component of HRQoL was lower (42.7±10.9 points; 95%CI 41.8–43.6) than mental component (48.0±7.9 points; 95%CI 47.3–48.6). Table 3 shows the means of both scores according to the sociodemographic and clinical variables. The physical component was lower in females and among the oldest age group, while those married/living with a partner showed a higher mean than the other categories. A more adverse socioeconomic situation (lower perceived economic situation, and lower economic position) negatively affected the physical HRQoL score, but the educational level was not associated with this outcome. The score was also lower amongst retirees or pensioners. A previous revascularization procedure was associated with better physical HRQoL, and both, an increased number of CVD comorbidities and a higher number of CVD risk factors adversely affected this outcome. In contrast, none of the sociodemographic or clinical variables was associated with the mental component of HRQoL (borderline p-values for gender, educational level, and perceived economic situation).

**Table 3:** Health related quality of life measured by SF-12 (physical and mental components) according to sociodemographic characteristics and clinical variables.

	Physical component		Mental component	
Variables	Mean	(95%CI)	Mean	(95%CI)
**Gender**		p = 0.033*		p = 0.091*
Male	43.2	42.2–44.2	48.3	47.6–49.0
Female	40.9	38.9–42.9	47.0	45.5–48.4
**Age group**		p = 0.001**		p = 0.360**
<70 years	43.9	42.3–45.5	47.2	46.0–48.4
70 to 79 years	43.2	41.9–44.5	48.7	47.8–49.5
≥ 80 years	39.6	37.7–41.4	47.7	46.2–49.2
**Marital status**		p = 0.002*		p = 0.099*
Married	43.5	42.5–44.5	48.3	47.6–49.0
Unmarried	40.3	38.4–42.2	47.0	45.6–48.5
**Residence area**		p = 0.534*		p = 0.119*
Urban	42.5	41.4–43.6	48.3	47.5–49.0
Rural	43.1	41.5–44.8	47.1	45.8–48.4
**State of general practice**		p = 0.627*		p = 0.435*
South Australia	42.5	41.3–43.7	48.2	47.4–49.0
Queensland	42.9	41.6–44.2	47.7	46.7–48.7
**Attained educational level**		p = 0.072**		p = 0.019**
Degree or higher	44.0	41.3–46.6	48.6	46.6–50.6
Certificate to advanced diploma	43.1	41.6–44.6	48.7	47.7–49.8
Secondary	42.5	41.1–43.9	47.5	46.5–48.5
No schooling to primary	40.5	37.9–43.2	46.2	44.1–48.3
**Working status**^a^		p<0.001†		p = 0.181†
Employed full or part time	47.6	45.6–49.6	47.8	46.3–49.4
Retired	44.0	42.3–45.7	49.3	48.2–50.4
Pensioner	40.9	39.7–42.1	47.6	46.7–48.4
**Perceived economic situation**		p<0.001**		p = 0.070**
Very high to higher	45.2	43.2–47.2	49.0	47.5–50.4
Similar to other people	43.1	41.9–44.2	48.1	47.3–48.9
Lower to very low	39.3	37.2–41.4	46.6	45.0–48.2
**Economic position (quintiles)**^a^		p = 0.040**		p = 0.477**
Highest	44.2	42.5–45.9	47.8	46.5–49.0
High	42.7	41.2–44.3	48.4	47.3–49.5
Middle	42.3	39.8–44.7	48.9	47.5–50.3
Low	41.6	40.0–43.3	47.2	45.9–48.5
**History of revascularization procedure**^b^		p = 0.009*		p = 0.272*
No	40.8	39.1–42.4	47.4	46.2–48.6
Yes	43.4	42.4–44.4	48.2	47.5–48.9
**Time since first ischaemic episode**^c^		p = 0.529**		p = 0.869**
Up to 5 years	43.0	41.6–44.4	48.1	47.0–49.1
6–10 years	42.9	41.1–44.6	47.9	46.7–49.1
10+ years	42.3	40.8–43.9	47.9	46.8–49.0
**Number of other CVD conditions**^d^		p<0.001**		p = 0.301**
0	44.3	43.3–45.4	47.6	46.8–48.4
1	41.3	39.5–43.1	48.7	47.4–50.0
2+	37.8	35.3–40.3	48.2	46.4–49.9
**Number of clinical CVD risk factors**^e^		P = 0.021**		p = 0.567**
0–1	44.3	42.5–46.1	47.7	46.3–49.1
2	42.9	41.5–44.3	48.6	47.6–49.7
3+	41.6	40.1–43.1	47.5	46.5–48.5

* T-test

** ANOVA test for trend

† ANOVA test for heterogeneity

CVD = cardiovascular disease

a–Based on the Socio-Economic Indexes for Areas Index of Relative Socio-economic Advantage and Disadvantaged (SEIFA-IRSAD). Individuals in the lowest quintile were not included due to logistic problems recruiting general practices in poorest areas.

b–Coronary angioplasty or coronary artery bypass graft

c–Angina or myocardial infarction diagnosis, or revascularization procedure

d—Atrial fibrillation/other arrhythmia, heart failure, stroke, heart valve disease, heart-block, cardiomyopathy, and/or left-ventricular dysfunction

e—Hypertension, overweight, dyslipidaemia, diabetes mellitus, and/or chronic kidney disease

Table 4 shows the crude and adjusted association between health literacy and HRQoL. Health literacy was directly associated with the physical component of HRQoL, with the extreme categories of health literacy showing a mean difference of 4.1 points (95%CI 1.4;6.8) for this outcome. When health literacy was incorporated in the regression model, the adjusted r^2^ increased from 13.7% (including just sociodemographic and clinical variables) to 15.3%. This corresponds to a 12% increase in the variability of the physical HRQoL score explained by the model (data not shown in Table 4). On the other hand, no association was observed between health literacy and the mental component of HRQoL in crude or adjusted analyses. In this case, the r^2^ remained stable at 3%, even after health literacy was included in the model. The mean VIF for both outcomes was 1.75, indicating no multicollinearity between the explanatory variables. No evidence of effect modification in the associations between health literacy and HRQoL was found according to age, gender, educational level, or socioeconomic condition (p-value of interaction >0.20 in all the cases).

**Table 4:** Crude and adjusted analyses of the association between health literacy and health related quality of life (physical and mental components).

	Health literacy			
Quality of life	Inadequate (n = 82)	Marginal (n = 289)	Adequate (n = 203)	p-value*
**Physical component**				
**Mean (95%CI)**	39.6 (37.1–42.1)	42.1 (40.8–43.3)	44.8 (43.3–46.2)	<0.001
**β crude (95%CI)**	-5.2 (-8.0;-2.4)	-2.7 (-4.7;-0.8)	Ref	<0.001
**β adjusted (95%CI)**^a^	-3.7 (-6.4;-1.0)	-2.9 (-4.8;-1.1)	Ref	0.001
**Mental component**				
**Mean (95%CI)**	47.6 (45.8–49.5)	47.8 (46.8–48.7)	48.5 (47.5–49.5)	0.274
**β crude (95%CI)**	-0.9 (-2.9;1.1)	-0.8 (-2.2;0.7)	Ref	0.274
**β adjusted (95%CI)**^b^	-0.5 (-2.6;1.5)	-0.6 (-2.0;0.8)	Ref	0.482

95%CI—confidence interval; β–regression coefficient

* P-values for trend

a–Adjusted for variables with p-value <0.10 in the association with the physical component of HRQoL: gender, age, age^2^, marital status, education level, working status, perceived economic situation, economic position (SEIFA-IRSAD quintiles), clinical history of revascularization procedure, number of other cardiovascular conditions, and number of risk factors for cardiovascular disease.

b–Adjusted for variables with p-value <0.10 in the association with the mental component of HRQoL: gender, age, age^2^, marital status, education level, perceived economic situation, and economic position (SEIFA-IRSAD quintiles).

## Discussion

The positive effect of health literacy on health outcomes has been widely discussed in the literature [8, 9, 11, 14, 15] but, to our knowledge, this is the first study to investigate its influence on HRQoL in patients with IHD. This relationship has been explained by poor adherence to recommended preventive approaches and treatment among patients with low health literacy, thus leading to poor disease control, and consequently affecting general health status [11, 12, 20, 30].

The direct association between health literacy and HRQoL among patients living with heart failure have been demonstrated in two cross-sectional studies in the United States [17, 20]. However, the comparability with these studies is limited, as disease-specific tools were used to evaluate HRQoL, which limits the capacity to assess the disease effect on general functioning. Additionally, these measurements provide a one-dimensional score, thus reducing the ability to discriminate the effects of health literacy on different components of HRQoL [21]. The SF12 is a short validated questionnaire to evaluate HRQoL, which has been proved to be robust when administered by different professionals and patient groups, with the benefit of providing information on physical and mental components of health status [28]. In our study health literacy was not associated with the mental component of HRQoL, a result that is consistent with other studies that also found that inadequate health literacy has a stronger effect on the physical component of HRQoL among patients living with chronic diseases [31, 32].

It has been reported that chronic conditions have a stronger effect on physical functional health status than on psychological functioning [33]. This also explains why the score for the physical component was lower than the mental component, although both were below the acceptable mean level (50 points) [29]. Consistent results were identified in a report of repeated surveys conducted in South Australia between 1997 and 2003 to evaluate population trends using the SF12 questionnaire. People living with heart disease (6.2% of the sample in 2003) had lower HRQoL scores compared to those without these conditions, with a stronger effect on the physical component (40.1±12.1 and 49.9±9.4, respectively) than on the mental component (51.7±10.7 and 52.3±8.5, respectively) [34]. These similarities suggest that the probability of bias in our study is low, even though the percentage of individuals who answered the HRQoL questionnaire was different according to gender, educational level, and socioeconomic status.

In high and middle-income countries CVD is the leading cause of morbidity and mortality, contributing significantly to health expenditures [1, 2]. Primary and secondary prevention have been proved to reduce the risk of CVD and its complications [5, 35, 36]. Adequate health literacy has been demonstrated as an important component of chronic disease management by reducing risk factors, recurrence, and further complications [17, 20, 37]. This could be explained because the long-term nature of chronic conditions, which requires participants to be knowledgeable and adhere to lifelong management of their condition [9]. Nevertheless, inadequate health literacy is still highly prevalent [13], with 60% of Australians classified as having inadequate health literacy in the last national report published in 2014 [12]. This frequency is four times the prevalence found in this study (14%). This difference is probably related to the characteristics of the target population in this study, as well as to the instrument used to measure health literacy, as other population-based research conducted in South Australia and Victoria found prevalence ranging from 21% to 25% when other validated instruments were used [38, 39]. In any case, these frequencies indicate a weakness in the management of patients with chronic conditions, especially when most healthcare professionals acknowledge that they lack the necessary skills to communicate with patients with limited health literacy [40].

Furthermore, this study also demonstrated that age was inversely associated with health literacy and with the physical component of HRQoL, which is consistent with studies investigating other chronic diseases [41, 42] and the general population [34]. Being elderly is associated with physiological and general health deterioration, including visual and hearing loss, which can also produce challenges for reading, listening, and understanding health information [41, 43]. Additionally, people of advanced age tend to have more than one chronic condition causing limitations on psychological and especially on physical functioning [6, 43], as evidenced in this study. We also expected that some indicators of broader health service utilization (history of revascularization procedure, longer time since first IHD episode, a larger number of CVD comorbidities and/or CVD risk factors) would have been related to better health literacy [44]. Nevertheless, none of these variables enhanced this characteristic, and furthermore, the number of CVD comorbidities adversely affected the prevalence of inadequate health literacy. These results are particularly worrying in public health terms, as a larger number of CVD comorbidities and CVD risk factors also decreased physical functioning, as confirmed in other studies [6, 43]. Therefore, this simultaneous adverse effect of CVD comorbidities on health literacy and HRQoL can compromise health management of patients with IHD, as almost 40% of them were diagnosed with at least one additional CVD condition.

A lower educational level was also associated with lower health literacy and reduced HRQoL, which is consistent with other results [9, 10]. Nevertheless, educational attainment cannot be used as an indicator of the level of health literacy, because even amongst individuals with high educational attainment there are still those with limited health literacy [39]. Individuals can still benefit from information through listening and seeing, even if they are unable to read or write [10]. In that sense, health practitioners may use a five-step process when providing care to the patients: 1) recognise consequences of low health literacy, 2) screen patients at risk, 3) document literacy levels, 4) document learning preferences, and 5) integrate effective strategies. These steps would enhance patients’ understanding into practice, thus increasing health literacy and improving health conditions [9].

A disadvantaged socioeconomic position also affected both, health literacy and the physical component of HRQoL. Socioeconomic status is an important social determinant of health, with disadvantaged individuals having lower health access, limited access to information, adverse lifestyle risk behaviours, and ultimately low health literacy [38, 45]. Additionally, some studies investigating HRQoL among patients with CVD also found a direct relationship between poor socioeconomic status and HRQoL [41, 46]. Nevertheless, it is noteworthy that in adjusted models the physical component HRQoL score was 5.9 points lower (95%CI -8.7;-3.0) in pensioner compared to employed participants, and the difference between the extremes of perceived economic situation was 4.6 points (95%CI -7.6;-1.5) (data not shown in tables). On the other hand, the score difference between the extreme categories of educational level or economic position was approximately 1.0 point in the adjusted analyses. This lack of effect for the last two covariates could be attributed to multicollinearity, but the VIF obtained was low. Therefore, working status and perceived economic situation were more important social determinants of the physical component HRQoL than educational level or economic position, showing a similar effect magnitude than the presence of other CVD. However, although a similar pattern was observed for the mental component of HRQoL, this outcome was not associated with the working status. These results are consistent with a systematic review published in 2013, which showed retirement having an adverse effect on general and physical health, but not on mental health [47]. Therefore, our results suggest the investigation of these socioeconomic characteristics, together with health literacy, and the coexistence of other CVD are extremely important in the assessment of patients with IHD to determine the risk of lower HRQoL and further complications.

Finally, females showed lower HRQoL (physical component) than males, but no association was found with health literacy. Nevertheless, evidence in the literature is contradictory about the direction of these associations [17, 39, 41, 48].

A limitation of this study is that patients in the lowest quintile of socioeconomic position were not included, which probably means we have underestimated the prevalence of inadequate health literacy and overestimated the mean of HRQoL. Nevertheless, regarding associations, such bias would mean that we have underestimated the real relationship between health literacy and HRQoL. Additionally, the sample size investigated in this study represents only 28% of the original target population (n = 2,132), with a reduced power to identify an association between health literacy and the mental score of HRQoL. Nevertheless, even, in that case, the study had enough power to identify a mean difference of at least 2.7 points between the categories of health literacy (~34% of the standard deviation), which is probably a value with clinical relevance. Finally, although representativeness could be a concern, no differences were observed between the individuals analysed in this study (n = 587) and those who answered just the first survey (n = 812) regarding any of the clinical variables or according to most of the sociodemographic characteristics. This suggests the likelihood of refusal bias in this study is probably low.

Our results suggest that the adverse effect of inadequate health literacy on the physical component of HRQoL may contribute to poorer physical functioning in patients with IHD. Therefore, the study highlights the importance of strengthening efforts to increase the level of health literacy across all patients with CVD, especially in older age groups, those with disadvantaged socioeconomic position (particularly among pensioners and those with a lower perceived economic situation), and among those living with other CVD. Also, when reviewing guidelines (national care and prevention guidelines), it is important to take into account different levels of health literacy. Practitioners should be conscious that inadequate health literacy and poor physical function are both higher in patients with IHD, which represent a barrier to effective care, and they should take this into account during every consultation. Improving health literacy and HRQoL would have not only direct effects on health conditions but also economic benefits to the individual and the health system.

## Supporting Information

S1 Dataset De-identified dataset including the variables used in this study (STATA v13 and CSV). (ZIP)
